# Alpha-gal Allergy in a 6-Year-Old Male: A Case Report

**DOI:** 10.31729/jnma.4557

**Published:** 2019-10-31

**Authors:** Masanosuke Kinoshita, Samuel Newton

**Affiliations:** 1 Theodosia Family Medical Clinic, Missouri, United States of America

**Keywords:** *allergy*, *food hypersensitivity*, *immunoglobulin E*, *meat products*, *tick bites*

## Abstract

Alpha-gal allergy is a reaction where the immunoglobulin E antibody elicits a response to galactose-alpha-l,3-galactose (alpha-gal) which is a mammalian oligosaccharide epitope found in nonprimate mammalian. After being exposed to a tick bite, particularly the Lone Star tick (Amblyomma americanum), an individual has been known to develop an alpha-gal allergy. Our patient presented with symptoms of delayed-onset allergy 3-8 hours after consuming mammalian meat products including beef and pork. These symptoms can include, but not limited to, the following: urticaria, angioedema, anaphylaxis, nausea, diarrhea, indigestion. Since symptoms do not present immediately, a delay in diagnosis can occur. Our patient highlights one of the few reported childhood cases with an alpha-gal allergy. We recommend a careful history, in particular, asking if the patient has a past history of tick bites and the appearance of allergy symptoms following beef or pork consumption.

## INTRODUCTION

Galactose-alpha-1,3-galactose (alpha-gal) allergy is an allergic reaction in an individual who has previously been exposed to a tick bite and subsequently develops allergic symptoms after consuming mammalian meat products, including beef and pork. Patients show symptoms of delayed-onset allergy 3-8 hours after eating beef or pork.^3,4^ These symptoms include urticaria, angioedema, anaphylaxis, nausea, diarrhea, indigestion. There have been many reports of adults with alpha-gal allergy, but very few reports on children.^1^ We present a case report on a young patient who presented with gastrointestinal symptoms at the initial presentation and then gradually developed a delayed allergic skin rash 11 months later.

## CASE REPORT

A six-year-old male living in Theodosia village, which is in the South Central area of Missouri, presented to the clinic with the chief complaint of abdominal pain, vomiting, and constipation for the past 3 weeks. Per the mother’s account, the patient had exhibited the abrupt onset of intermittent colicky abdominal pain. The patient also had been experiencing constipation and difficulty with bowel movements. When the patient did have bowel movements, the mother described the stool to be soft and jelly-like. In addition, the patient had been vomiting after eating a meal resulting in a decrease in his appetite. The patient continued to hydrate. The patient denied eating new foods or developing any rash eruptions. The patient describes the pain as pressure that comes and goes, and he rated the pain as 6 out of 10.

During the physical examination, the patient’s abdomen was found to be soft, non-tender, and non-distended in all four quadrants. There was no palpation of a solid mass. The patient’s bowel sounds were found to be positive. There was no detection of hepatosplenomegaly.

Based on the description of the patient’s stool as “jelly” in appearance, in conjunction with intermittent abdominal pain and vomiting, we initially thought that the patient might be experiencing an intussusception. Abdominal ultrasound and stool culture were ordered. Our initial dietary recommendations were to eat plain foods such as bananas, papaya, yogurt, rice, crackers, and continue oral fluid hydration. Subsequently, the patient’s abdominal symptoms improved after a few days, and close observation was recommended. Ultrasound of the complete abdomen was reported normal for a 6-year-old male. Stool cultures revealed no detection of pathogens or toxins.

Eleven months later, the patient now seven years old, returned to the clinic with the chief complaint of a rash erupting diffusely across the body. The patient’s mother described her son as coming home after playing in the woods and developing a rash later that night before going to bed. The rash appeared itchy and irritating. The patient’s mother had provided her son with an over the counter diphenhydramine medication; however, there was little improvement. The rash spread from the upper/lower extremities, chest, and back. The patient’s face was spared. The patient’s mother denied using new laundry detergents or bath soaps. The patient also denied consuming new food or beverages. The patient reported that he had eaten a hamburger and a salad the evening prior to the eruption of the rash.

**Figure 1:**
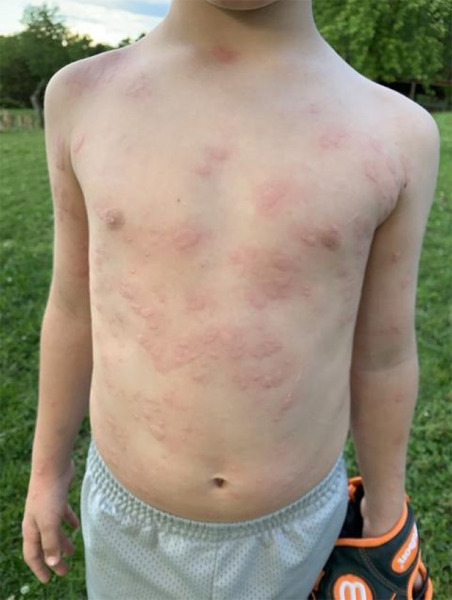
Photograph of the patient with allergic rash.

During the physical examination, several asymmetric oval erythematous, raised maculopapules were found across the patient’s extremities and torso (Figure 1).

The rash was non-tender but pruritic to touch. The rest of the physical exam, including the patient’s abdomen, was within normal limits. The patient’s mother reported that she had noticed that the patient had complained of significant abdominal pain and diarrhea after eating his hamburger. In retrospect, the patient apparently was having these symptoms for the past 11 months.

Based on the new chief complaint of a pruritic rash eruption after eating beef, and the history of abdominal pain with diarrhea after eating a meat product, the correlation suggested a possible diagnosis of an alpha-gal allergy. The patient also lives in an area where tick bites are common, which would provide the vector for developing an alpha-gal allergy. Other differential diagnoses would include contact dermatitis or an allergic reaction to a food or beverage. Follow-up labs included a complete blood count, complete metabolic panel, and alpha-gal antibody titer. The patient was prescribed hydrocortisone cream and advised to continue using over the counter diphenhydramine for itching, and calamine lotion to be applied over the rash. Subsequently, the patient’s laboratory results returned, indicating a positive result for alpha-gal immunoglobulin E (IgE). The patient and the patient’s mother were informed to avoid meat products, including beef and pork. The patient’s symptoms gradually improved a few days later.

## DISCUSSION

Alpha-gal allergy occurs when the immune system develops an IgE antibody toward alpha-gal carbohydrates. Alpha-gal is present in all non-primate mammals.^1^ Primate mammals including; apes, Old World or Cercopithecoidea monkeys, and humans, which do not naturally possess alpha-gal.^1^ In addition, alpha-gal is found in the immunoglobulin G (IgG) monoclonal antibody cetuximab, which is used to treat cancer of the head, neck, and colorectum.^2^ Thomas Platts-Mills reported to have been the first to identify the alpha-gal allergy in the United States during the early 2000s.^1^ Platts-Mills and his associates recognized the similarities of patients reporting allergies to cetuximab and to patients reporting allergies to meat consumption in the Southeastern United States. Moreover, they recognized the role of the Lone Star tick (Amblyomma americium) bite that lead to individuals developing an allergy to meat after 3 members of their own research group presented with allergy symptoms. They subsequently discovered the distribution of tick bites overlapping with the patients reporting allergies to meat.^1^

The currently accepted theory of how an individual develops an alpha-gal allergy is that ticks carry residual glycoproteins, or glycolipids, with the alpha-gal epitope, in their saliva. Upon biting a human, the alpha-gal enters the bloodstream. The immune system will then develop an IgE response. Future consumption of red meat will then cause an individual to experience an alpha-gal allergy reaction.

The symptoms of alpha-gal allergy are urticaria, angioedema, anaphylaxis, nausea, abdominal pain, diarrhea, and indigestion. These symptoms can appear between 3-8 hours after consuming meat.^3,4^ There is a delay of these symptoms due to the time-lapse of absorption of alpha-gal protein/sugar entering the circulation from the gastrointestinal tract.^3^ At present, alpha-gal is diagnosed by performing immunologic testing for alpha-gal IgE. An IgE level that is greater than 0.35 kU/L is considered positive.^2^ Skin prick allergy testing has been found to give false negative result.^2^

Due to the delay in the presentation of symptoms, patients may not make the association of symptoms following the consuming red meat. From a clinical perspective, a red meat allergy has not been readily associated with a past tick bite. A clinician would need to be cognizant of the correlation of allergic symptoms and the prior consumption of red meat. A history of a previous inflamed tick bite may be a supportive clue. Tick bites are very common and generally cause local inflammation, so any particular tick bite may not raise suspicion.

There is currently no known cure for alpha-gal allergy. Patients should avoid being bitten by ticks by wearing clothing that minimally exposes skin and the use of tick repellant products. When diagnosed with alpha-gal allergy, patients should restrict the consumption of mammalian meat, including beef, lamb, and pork. It may also be best to avoid consuming all products derived from the mammalian. For example, galpha-gallatin, milk, or cheese.^4^ An antihistamine and cortisone may be provided to treat the rash, swelling, and pruritus. An epinephrine autoinjector (EpiPen) should be provided to a patient for a potential anaphylactic reaction.

So far, in the United States, only the Lone Star tick bite has been identified to elicit the alpha-gal allergy. In Australia, the Ixodes holocyclus tick has been noted to cause alpha-gal allergy, while in Europe, the Ixodes Ricinus has been classified as a contributing vector. In Asia, such as Japan and South Korea, tick bites and the subsequent development of meat allergies have also been reported.

Due to alpha-gal allergy symptoms appearing in a delayed fashion, arriving at this correct diagnosis may be difficult. The mean time for diagnosis after the patient first reports symptoms is said to be 7.1 years.^2^ Patients are often dismissed with a diagnosis other than alpha-gal allergy. Due to very few published case reports on alpha-gal allergy, clinicians may not be aware of the clinical presentation.^1,2^ Furthermore, there are very few published reports on children.^1^

Our case report highlights alpha-gal allergy in a young patient. Our patient may have developed an alpha-gal allergy 11 months prior to the time he complained of gastrointestinal symptoms. The development of the urticarial rash followed the presentation of the abdominal symptoms. For healthcare providers, it is important to keep alpha-gal as one of the differential diagnoses for a patient living in areas with high tick prevalence. As alpha-gal is an IgE mediated allergic response, life-threatening anaphylaxis can occur, although delayed for 3-8 hours after consuming meat.^3,4^ Our patient was diagnosed with alpha-gal only after careful history taking and recognizing that there was previous exposure to a tick bite, and that symptom occurred after consuming red meat. Therefore, it is important to better understand and recognize alpha-gal allergy.
